# Hypertrophied Tonsils and Adenoids an Etiological Factor in Backward Children

**Published:** 1910-09

**Authors:** Geo. H. Cooper

**Affiliations:** Opelika, Ala.


### HYPERTROPHIED TONSILS AND ADENOIDS AN ETIOLOGICAL FACTOR IN BACKWARD CHILDREN.*

BY GEO. H. COOPER, M. D., OPELIKA, ALA.

Every physician will concede the fact that there is no single condition, with perhaps the exception of heredity, that plays so important a part in retarding the normal growth and development, both mentally and physically, of the infant and young child, as does hypertrophy of the faucile tonsils and adenoids. This fact is so universally recognized that I feel an apology is due my hearers for asking your attention to a paper of this character. The subject is of vital importance, much good may come from a free discussion of it, and possibly by this means, we may be able to influence some of our fellow-practitioners to be on the lookout for adenoids in infants, even when the tonsils are not enlarged.

That adenoids very often exist without enlarged tonsils is not universally recognized. Snuffles are too often attributed to colds in the head, in the great majority of the cases we will find that the child has adenoids. When we do find them, we ought not to temporize with sprays and washes, but should advise their removal with the curette, which is best done under general anaesthetic. The hemorrhage is not profuse and the next day the snuffles and cold in the head are gone. However, the operation can be done successfully without a general anaesthetic. A few strokes with the hand will quickly clear the posterior nares.

The relation of this subject to the family doctor is of great importance. There is yet a remnant of the generation of doctors who ignorant of the irreparable damage done to the mouth, throat and delicate structures of the ear during the years of the growing child, discourage operation, advising that the child will outgrow the trouble. It is within the domain of the family physician to advise and direct the care of children in such a manner that they will grow up vigorous and healthy offspring, with prospects of having a healthy state physically, mentally and normally, and become useful members of society. We must teach the parents that if there is any difficulty in breathing during the first few weeks, or months, of infant life, they must consult their physician. We must also teach them that the normal child nurses well, sleeps well, increase steadily in weight, and is not fretful either in its waking or sleeping hours, and we must impress upon the minds of the parents that when the child has these abnormal conditions, it is more susceptible to contagious diseases and that its vitality will become markedly impaired, thereby making it more liable to contract the infectious disease, such as tuberculosis, typhoid fever and intestinal disturbances.

When we explain to the parents that the operation can be done without a general anaesthetic (this being the greatest dread of them), that it takes only a moment, that the child will not be confined to bed, that it will surely sleep better, eat better, and develop faster in every way from the moment it is operated upon, then not one parent in fifty will oppose the operation.

The Health Department of New York in 1905, began a systematic examination of all school children. These examinations were especially for defective hearing, vision and diseases of the respiratory organs. Nearly 70 per cent. of these children possessed abnormalities which could be easily corrected, but which, if neglected, would make them fit subjects for institutions for defectives and delinquents. In one school alone 90 per cent. were operated upon for enlarged tonsils and Adenoids. This was a special school for backward children, and a report from 80 per cent. of them three years later, showed that they had done better school work in the subsequent six months than they had been able to do in two years previous to the operation.

Had not this inspection been carried on, and had nothing been done for these children, they would not have been benefitted for study or any important position in after life, whereas now they will become valuable members of their respective communities. Many of these children had defective noses and throats from early infancy and little attention was paid to them. When these children became old enough to enter school they did not learn as normal children, seemed lazy, dull, stupid, obstinate and rebellious, necessitating their removal to a special school for backward children. Here the true condition was discovered and corrected with the miraculous results stated. In systematic examination of school children, diseased noses and throats would not be the only abnormal conditions found.

It would be discovered that a great percentage had defective eyesight, impaired hearing, deformed chest, poor digestion, wrecked nervous system, rheumatism, rickets and were slow to learn, etc.

Some parents argue that they themselves had enlarged tonsils and outgrew them, while as a matter of fact, they did not outgrow them but the relief derived as years advanced was due to the growth of the pharynx rather than the atrophy of the gland. In many cases of enlarged tonsils and adenoids a true fibrosis exist, and when a true fibrosis does supervene, atrophy seldom if ever takes place, and the condition consequently persists until corrected by operation.

Out of 2,000 New York school children 30 per cent. possessed Adenoids.

### Etiology.

Enlarged tonsils and Adenoids are diseases of childhood and young adult life. In many instances the condition subsides as the child reaches maturity, but in waiting for that time to come irreparable damage may be done, and many of the most important years of the child's life lost, for it is during these years that the foundation of the child's future health is begun. This predisposition in childhood to enlarged tonsils and Adenoids is probably due to the high activity of the epethelial and lymphatic structures, that is, a child seems to possess a lymphatic temperament so that, when kept in too close quarters, or when there is not sufficient fresh air in the sleeping room, it gets a marked congestion of the mucous membrane of the air passages as soon as it is taken out of doors. This being often repeated produces a swelling of the mucous membrane and lymphatics, with a resultant obstruction to the free passage of air, and still later a true pathologic condition of all the structures in the nasopharynx.

Heredity, diphtheria, scarlet fever, measles and other debilitating systemic diseases play an important role in the etiology of enlarged tonsils and Adenoids.

### Symptomatology.

Probably the most troublesome symptom of these growths is the excessive secretion of mucous in the posterior nares. We all have seen them, especially among the neglected classes, their posterior nares completely blocked by this mucus, and large quantities draining from the anterior nares, producing a very repulsive condition of the face, to say the least. This mucus forms hard crusts which further obstruct the child's breathing, and also prevents the secretion from dropping into the lower throat, where it could do little harm. Instead it is retained in the nose, degenerates into mucopus, becomes highly infected by pathologic organisms from the atmosphere and eventually lodges in the crypts and fissures of the naso-pharynx, becoming a hot-bed for the further development of disease germs. Finally, it passes into the stomach or lungs, where the organisms multiply, still more rapidly, until we have a chronic gastritis, acute tuberculosis or intestinal infection to deal with.

The child with this condition now develops an altered voice, a "nasal twang," that is, the child is said to talk through its nose as if it had a "cold in its heads," whereas in fact, the voice becomes so because the child cannot talk through its nose. Parents will often bring forward the objection of the operation that; "the child will not be able to sing as well if the tonsils are cut"; they do not understand that the abnormal condition will do that very thing unless corrected and that we aim by operation to put the throat and nose back into the same condition that nature intended it to be. No normal structures are removed, only those that are diseased and so swollen that they act as foreign bodies and prevent the child from singing and talking clearly. This obstruction to free breathing soon produces a peculiar facial expression characteristic of adenoids. The child develops drooping eyelids, broadening of the nose, drooping of the lower jaw and lip. In fact, all the bones and muscles of the face fall into disuse, and produce a vacant semi-idiotic expression, so that all who come in contact with the child think that it is not possessed of all its wits.

One of the most interesting problems in medicine today is the possible prevention of aural diseases in adult life, by removal of the causes in early childhood. It is universally acknowledged by the medical profession that the dominant factor in middle ear disease is infection, and this infection could come from no other source than through the Eustachian tubes, with the possible exception of metastasis, as McKenzie claims that the discovery of adenoids has saved thousands of cases of deafness every year.

Every child at the Central London Nose, Throat and Ear Hospital is submitted to Adenectomy. They also claim that adenoids are responsible for a large per cent. of deafness.

A chronic cough of greater or less severity is a fairly constant symptom of enlarged tonsils and Adenoids.

One of the most important functions of the mucous membrane of the nares is to render the air humid as it passes to the lungs, but when the air is breathed in through the mouth it extracts the natural moisture of the larynx trachea and bronchi; keeping mucous membrane dry and irritated and producing a dry rasping cough, with its consequent debilitating influence upon the general health and resultant reflex nervous symptoms. It may now be easily seen how readily tuberculosis infection may take place. It is a well established fact that many tuberculous patients state that the trouble was preceded by a chronic catarrhal inflammation of the throat and nose.

There is no question in my mind had the naso-pharynx of many tuberculosis patients been in healthy condition they would have never had tuberculosis.

### Diagnosis.

The catarrhal discharge, the facial expression and a history of disturbed sleep will usually enable us to make a diagnosis. This may be confirmed by posterior Rhinoscopy and the finger. Very soon after the operation the child feels better, so much better that it seems as if nature was delighted to get rid of the offending material. The child acts as if a great burden had been lifted from its shoulders; the flesh becomes pink and firm, the eyes regain their lustre, the face assumes a happy, intelligent expression, hearing becomes more acute, the child sleeps better, learns more rapidly and grows faster.

Hypertrophic and atrophic rhinitis, headache asthma, epistaxis, nightmare, night sweats, convulsions, rickets, anemia, pigeon breast, round shoulders, pallor, vertigo, sneezing, loss of smell and taste, aphonia, salivation, stammering, chorea, rheumatism, heart disease and indigestion are common sequelae.

Surely there is no other act that we can do in our professional capacity that wins such praise and gratitude from the parents of these little ones as the removal of diseased Adenoids, and tonsils.

